# Magnetic Field Effects on the Structure, Dielectric and Energy Storage Properties of High-Entropy Spinel Ferrite (La_0.14_Ce_0.14_Mn_0.14_Zr_0.14_Cu_0.14_Ca_0.14_Ni_0.14_)Fe_2_O_4_/PVDF Nanocomposites

**DOI:** 10.3390/polym15081842

**Published:** 2023-04-11

**Authors:** Jiale Qiao, Haiwei Mu, Chao Liu, Zhaoting Liu

**Affiliations:** 1 School of Physics and Electronic Engineering, Northeast Petroleum University, No. 199, Fazhan Road, Daqing 163318, China; 2 School of Electrical Engineering, Suihua University, Suihua 152001, China

**Keywords:** dielectric constant, energy storage, high-entropy materials, PVDF-nanocomposites

## Abstract

Energy depletion is one of the significant threats to global development. To increase the usability of clean energy, the energy storage performance of dielectric materials must be urgently enhanced. Semicrystalline ferroelectric polymer (PVDF) is the most promising candidate for the next generation of flexible dielectric materials thanks to its relatively high energy storage density. In this work, high-entropy spinel ferrite (La_0.14_Ce_0.14_Mn_0.14_Zr_0.14_Cu_0.14_Ca_0.14_Ni_0.14_Fe_2_O_4_) nanofibers (abbreviated 7FO NFs) were prepared by the sol-gel and electrostatic spinning methods, then blended with PVDF to prepare composite films using the coating method. A magnetic field was used to control the orientation distribution of the high-entropy spinel nanofibers in the PVDF matrix. We investigated the effects of the applied magnetic field and the content of high-entropy spinel ferrite on the structure, dielectric, and energy storage properties of the PVDF substrate films. The 3 vol% 7FO/PVDF film treated in a 0.8 T magnetic field for 3 min exhibited a good overall performance. The maximum discharge energy density was 6.23 J/cm^3^ at 275 kV/mm and the efficiency was 58% with 51% β-phase content. In addition, the dielectric constant and dielectric loss were 13.3 and 0.035, respectively, at a frequency of 1 kHz.

## 1. Introduction

**Figure 1:**
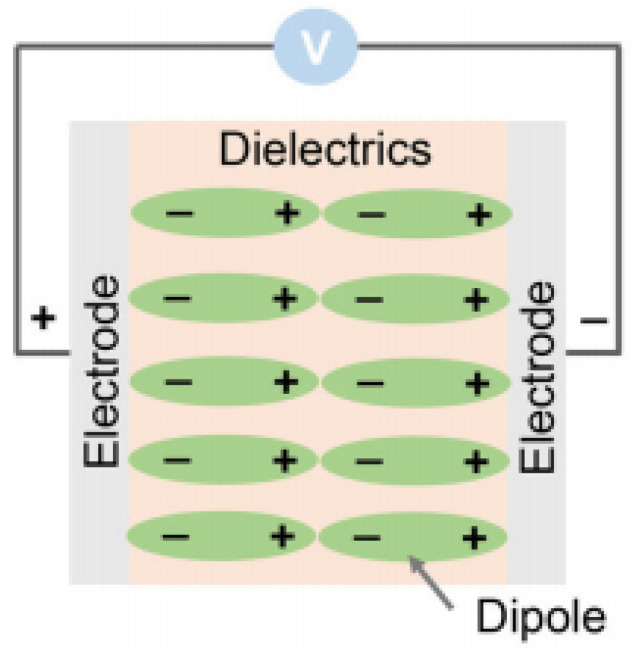
Energy storage mechanism of electric dipoles.

The rapid development of clean energy sources such as solar, wind, hydroelectric, and nuclear power provide effective solutions to major global energy crisis problems, including non-renewable resource scarcities (e.g., petroleum, coal, etc.) and environmental pollution. The full utilization of clean energy requires the integration of advanced energy storage technologies to overcome intermittency, inefficiency, and expensive inputs [1,2]. Benefiting from the physical storage mechanism of an electric dipole, as shown in Figure 1 [3], dielectric energy storage capacitors offer ultra-high-power density (megawatts), ultra-fast response time (milliseconds), low losses, long lifetimes cycle (>$10^{5}$ times), and high operating voltage (MV/m). They are the preferred alternative to electrochemical devices such as supercapacitors and lithium batteries [4]. Therefore, they are rapidly gaining attention in new energy development as well as in the field of energy storage. In particular, dielectric energy storage capacitors are good candidates for future miniaturization and high-efficiency energy storage materials due to their mechanical flexibility, lightweight design, easy processing ability, low cost, and many other irreplaceable advantages.

To date, five typical dielectric materials have been extensively studied: ferroelectrics (FE), paraelectrics (PE), relaxed ferroelectrics (RFEs), antiferroelectrics (AFE), and linear dielectrics (LD) [5,6,7,8]. Among these materials, ferroelectric PVDF polymers are attractive and flexible energy storage materials thanks to their high breakdown strength, light mass, ease of preparation, and high energy storage density [9,10].

Typical capacitors use a sandwich architecture with two horizontal layers of metal electrodes coated on the outside and a dielectric material sandwiched between them. When an external electric field is applied at both ends of the electrodes, the middle dielectric layer is polarized, the dipoles are elongated and aligned, and the charges accumulate at the electrodes on both sides, creating an internal electric field. In this way, the electrostatic energy is stored in the capacitor. When the capacitor is connected to an external circuit, the dielectric layer is depolarized and the stored energy is released [11]. Therefore, the performance of a capacitor is mainly determined by the dielectric material. The discharge energy density ($U_{discharge}$) of the dielectric material can be calculated by integrating the potential shift (*D*) in the electric field (*E*) loop. Then, the discharge energy density can be determined by [12], as shown in Equation (1):

(1) $$U_{discharge}=\int_{Dmin}^{Dmax}EdD$$

where, *D*, *E*, and $U_{discharge}$ represent the electric displacement, electric field, and discharge energy density (sometimes called the recoverable energy density), respectively. *D* and polarization (*P*) are related as follows: $D=\varepsilon_{0}\varepsilon_{r}E=\varepsilon_{0}E+P$, where $\varepsilon_{0}$ is the vacuum dielectric constant, the value $\varepsilon_{r}$ is the relative dielectric constant pertaining to the material’s properties, and P represents the polarization. The energy storage efficiency can be calculated by the following equation: $U_{discharge}/\left(U_{discharge}+U_{loss}\right)$. For linear dielectrics, the discharge energy density can be expressed as $U_{discharge}=1/2\varepsilon_{0}\varepsilon_{r}Eb^{2}$. For a high dielectric constant, the dielectric medium material *P* is approximately equal to *D*, meaning that the discharge energy density can be rewritten as $U_{discharge}=\int_{Pr}^{Pmax}EdP$. Here, Pr represents the residual polarization after discharge; the smaller the value, the higher the energy storage efficiency. Excellent energy storage performance often requires large polarization and small residual polarization.

Popular methodologies to boost energy storage in dielectrics include domain modulation [13,14], physical field processing [4], topology modulation [15,16], multilayer films [11], atomic-level defective design [17], all-organic polymer co-blended membranes [18,19], gradient films [20,21], high dielectric ceramic/polymer nanocomposites [12,22], and more. Polymer-based nanocomposites are used to improve energy storage performance by increasing the dielectric constant and breakdown strength through doping and other means. Recently, high-entropy materials have attracted the attention of scholars for having at least four advantages [23,24], namely, phase stability, atomic disorder with lattice distortion, slow diffusion dynamics, and the cocktail effect [25,26]. The first demonstration of phase-stable high-entropy oxides was made in 2015 [27], and subsequent attempts have been made in the literature involving high-entropy materials with high dielectric constants and low dielectric losses associated with dielectric properties [28,29]. Entropy can be defined in two ways, as described below.

In thermodynamics, the phase stability of HECs is governed by the Gibbs free energy, which, as shown in Equation (2), can be calculated by defining

(2) $$\Delta G_{mix}=\Delta H_{mix}-T\Delta S_{mix}$$

where $H_{mix}$ is the mixing enthalpy and $S_{mix}$ is the mixing entropy, which can be expressed by [30], as shown in Equation (3):

(3) $$\Delta S_{conf}=-R\sum_{x_{i}}\left(x_{i}\sum_{n}c_{j}\ln c_{j}\right)$$

where *n*, *R*, $c_{j}$, and $x_{i}$ are the number of components, gas constant, atomic fraction of components *j*, and fraction of the sublattice *i*, respectively. Higher entropy facilitates a lower Gibbs free energy, resulting in better phase stability.

Sarker et al. [31] proposed a descriptor of entropy forming ability (EFA) that uses the firstness principle in combination with the Boltzmann distribution and the degeneration of the state to stochastically compute the energy distribution spectrum; in this way, it is possible to capture the accessibility of isosampled states in the vicinity of the ground state and to quantify the conformational disorder that can stabilize the high-entropy homogeneous phase, as shown in Equations (4) and (5):

(4) $$EFA={\left(\sqrt{\frac{\sum_{i=1}^{m}g_{i}{\left(H_{i}-H_{mix}\right)}^{2}}{(\sum_{i=1}^{m}g_{i})-1}}\right)}^{-1}$$

(5) $$H_{mix}=\frac{\sum_{i=1}^{m}g_{i}H_{i}}{\sum_{i=1}^{m}g_{i}}$$

where *m* is the sum of the sampled geometric configurations, $g_{i}$ is their simplicity, and $H_{mix}$ is the enthalpy of the mixed phase, which is approximated by averaging the enthalpy of the sampled configurations $H_{i}$.

Adding magnetic materials to a ferroelectric polymer base produces a magnetic dielectric effect, which in turn affects the dielectric constant of the polymer base [32,33,34,35]. However, to the best of our knowledge, there is little information on doping high-entropy spinel ferrite nanofibers (La_0.14_Ce_0.14_Mn_0.14_Zr_0.14_Cu_0.14_Ca_0.14_Ni_0.14_)Fe_2_O_4_ in PVDF base with an applied magnetic field to modulate the orientation arrangement of high-entropy spinel ferrite 7FO NFs in a PVDF base. The effects of magnetic field treatment on the film material structure and the dielectric and energy storage properties of 7FO/PVDF nanocomposite are largely unknown. We propose that a magnetic field and doping with high-entropy materials can be used to optimize the dielectric and energy storage properties of PVDF-based materials.

## 2. Materials and Methods

### 2.1. Materials

$C_{6}H_{8}O_{7}H_{2}O$ was purchased from Tianjin Tianda Chemical Reagent Factory, while polyvinylidene fluoride (PVDF) powder was purchased from Shanghai 3F New Material Co., Shanghai, China. N,N-dimethylformamide (DMF), $\mathrm{Cu}{\left({\mathrm{NO}}_{3}\right)}_{2}3H_{2}O,\;\mathrm{Ni}{\left({\mathrm{NO}}_{3}\right)}_{2}6H_{2}O,\;\mathrm{Ca}{\left({\mathrm{NO}}_{3}\right)}_{2}4H_{2}O$, and anhydrous ethanol $\left(C_{2}H_{6}O\right)$ were obtained from Sinopharm Chemical Reagent Co., Shanghai, China. $\mathrm{Fe}{\left({\mathrm{NO}}_{3}\right)}_{2}9H_{2}O$, while ${\mathrm{CeN}}_{3}O_{9}6H_{2}O$ was purchased from MACKLIN. ${\mathrm{MnN}}_{2}O_{6}4H_{2}O$, $\mathrm{Zr}{\left({\mathrm{NO}}_{3}\right)}_{4}5H_{2}O$ and ${\mathrm{LaN}}_{3}O_{9}6H_{2}O$ were purchased from Aladdin. All chemicals or reagents were of analytical purity and required no further processing.

### 2.2. Synthesis of High-Entropy Spinel Ferrite (La_0.14_Ce_0.14_Mn_0.14_Zr_0.14_Cu_0.14_Ca_0.14_Ni_0.14_)Fe_2_O_4_ Nanofibers

**Figure 2:**
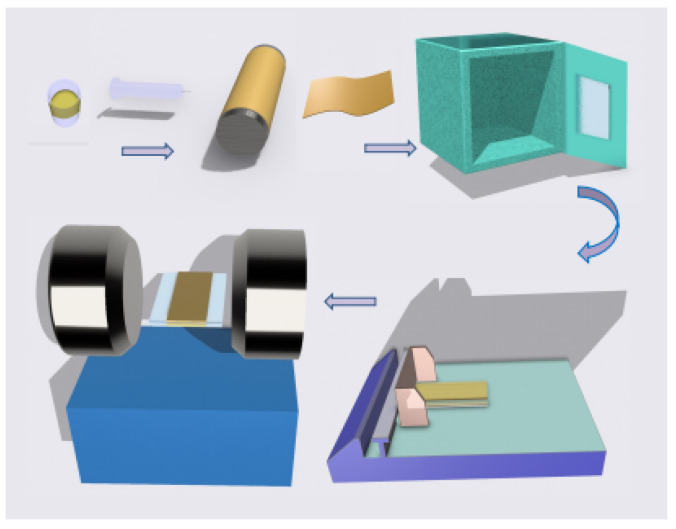
Preparation process of (La_0.14_Ce_0.14_Mn_0.14_Zr_0.14_Cu_0.14_Ca_0.14_Ni_0.14_)Fe_2_O_4_/PVDF films.

High-entropy spinel ferrite (La_0.14_Ce_0.14_Mn_0.14_Zr_0.14_Cu_0.14_Ca_0.14_Ni_0.14_)Fe_2_O_4_ nano-fibers were prepared by the sol-gel and electrostatic spinning methods [36,37,38]. The main steps in the synthesis process of 7FO NF are shown in the upper part of Figure 2. First, using the sol-gel method, seven hydrated nitric acids (with a total molarity of 0.1 mol, keeping the same molarity of each nitric acid), 0.2 mol of $\mathrm{Fe}{\left({\mathrm{NO}}_{3}\right)}_{3}9H_{2}O$, and an appropriate amount of citric acid $C_{6}H_{8}O_{7}$ were weighed and dissolved in 6 mL of deionized water and 20 mL of anhydrous ethanol in separate portions. The co-mixture was stirred vigorously for 6 h. After that, 2 g of PVP was added to the clear solution and stirred for 6 h to obtain the precursor solution. Then, a syringe with a metal needle filled with the precursor solution was installed on the electrostatic spinning machine using the electrostatic spinning method. The working voltage was 20 kV, and the distance between the needle and the receiver was set to 15 cm. Only amorphous mixed fibers were obtained on the roller receiver. To obtain the spinel structure of 7FO, we calcined the amorphous NF composite in a muffle furnace at 600 °C for 3 h.

### 2.3. Fabrication of 7FO/PVDF Nanocomposite Film Materials

As shown in the lower part of Figure 2, B 1 vol%, B 3 vol%, B 5 vol%, B 7 vol%, B 10 vol%, 1 vol%, 3 vol%, 5 vol%, 7 vol%, and 10 vol% 7FO NFs of PVDF-based composite films were prepared using the conventional solution casting method. Here, B represents the magnetic field. A magnetic field strength of 0.8 T was applied to the film for 3 min. The magnetic field direction was parallel to the film plane.

First, 7FO NF was weighed and added to 30 mL of DMF solution at different volume contents (1, 3, 5, 7, and 10 volume % of NFs). After that, PVDF powder was dispersed in the above suspension in small amounts several times and stirred for 12 h to ensure that it completely dissolved in the DMF. After resting and slowly pouring on a clean glass substrate, the film was coated onto the coating machine and the coated film was placed in the magnetic field or drying oven for post-processing as fast as possible.

### 2.4. Characterization

The 7FO NFs and 7FO/PVDF nanocomposite films were measured using a Rigaku SmartLab SE X-ray diffractometer (XRD) for crystal structure with a 2θ angle test of 10–80${}^{\circ }$. A field emission scanning electron microscope (SEM, Hitachi S4800, Hitachi, Tokyo, Japan) was used to collect the microscopic morphology information of both, and an Oxford X-Max device was used to test the energy spectrum for compositional analysis and face scanning. The functional groups of the fillers and composites were characterized using a Thermo Scientific Nicolet 6700 FTIR (Fourier Transform Infrared Spectrometer), New York, NY, USA.

The films were coated with metal Ag electrodes on both sides prior to testing the electrical properties. The dielectric properties were measured using a broadband dielectric spectrometer (model Alpha-A, Novocontrol, Montabaur, Germany) at $10^{2}$ Hz to $10^{7}$ Hz. The ferroelectric properties were measured using a Radiant Premier II ferroelectric test system with a D-E hysteresis line at 10 Hz. Finally, the discharge energy density and discharge efficiency were calculated from the hysteresis loop.

## 3. Result and Discussion

**Figure 3:**
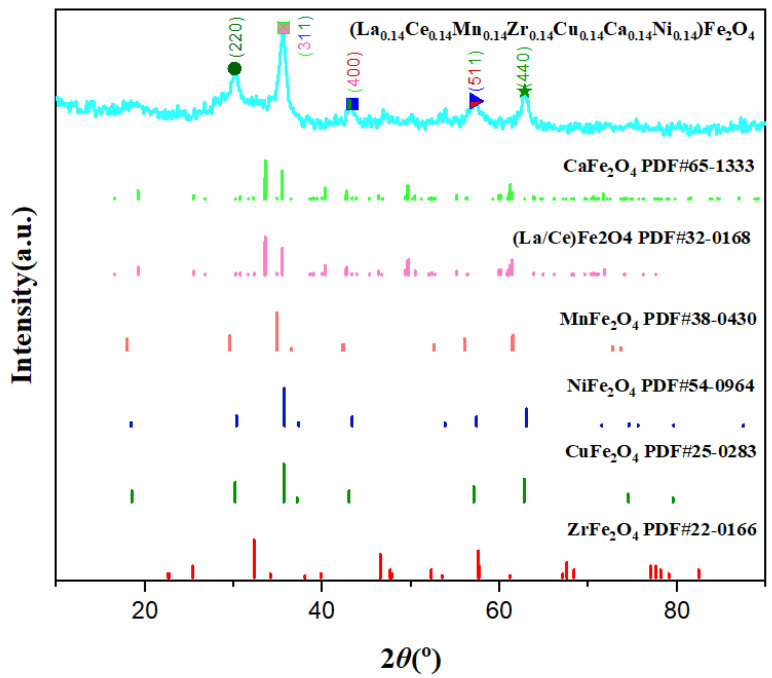
XRD curve of (La_0.14_Ce_0.14_Mn_0.14_Zr_0.14_Cu_0.14_Ca_0.14_Ni_0.14_)Fe_2_O_4_ NFs.

The composition and morphology of the high-entropy spinel ferrite were investigated by XRD, SEM, and EDS Mapping. Figure 3 shows the XRD patterns of (La_0.14_Ce_0.14_Mn_0.14_Zr_0.14_Cu_0.14_Ca_0.14_Ni_0.14_)Fe_2_O_4_ NFs and the standard PDF card patterns of ${\mathrm{CuFe}}_{2}O_{4}$, ${\mathrm{NiFe}}_{2}O_{4}$, ${\mathrm{CaFe}}_{2}O_{4}$, ${\mathrm{CeFe}}_{2}O_{4}$, ${\mathrm{MnFe}}_{2}O_{4}$, ${\mathrm{ZrFe}}_{2}O_{4}$, and ${\mathrm{LaFe}}_{2}O_{4}$. Each characteristic peak of the high-entropy spinel ferrite matched the characteristic peaks of the spinel ferrite containing the element. For example, the five distinctive characteristic peaks when 2θ was 30.1${}^{\circ }$ correspond to the (220) crystal plane of ${\mathrm{CuFe}}_{2}O_{4}$, ${\mathrm{NiFe}}_{2}O_{4}$, and ${\mathrm{MnFe}}_{2}O_{4}$; 35.5${}^{\circ }$ corresponds to (311) crystal plane of ${\mathrm{CuFe}}_{2}O_{4}$, ${\mathrm{NiFe}}_{2}O_{4}$, ${\mathrm{CaFe}}_{2}O_{4}$, ${\mathrm{CeFe}}_{2}O_{4}$, ${\mathrm{ZrFe}}_{2}O_{4}$, and ${\mathrm{LaFe}}_{2}O_{4}$; 43${}^{\circ }$ corresponds to (400) crystal planes of ${\mathrm{CaFe}}_{2}O_{4}$, ${\mathrm{CeFe}}_{2}O_{4}$, ${\mathrm{MnFe}}_{2}O_{4}$, and ${\mathrm{LaFe}}_{2}O_{4}$; 57${}^{\circ }$ corresponds to (511) crystal planes of ${\mathrm{NiFe}}_{2}O_{4}$ and ${\mathrm{ZrFe}}_{2}O_{4}$; and 63${}^{\circ }$ corresponds to (440) crystal planes of ${\mathrm{CuFe}}_{2}O_{4}$. Thus, the synthesized spinel ferrite contains La, Ce, Mn, Zr, Cu, Ca, Ni, and Fe.

**Figure 4:**
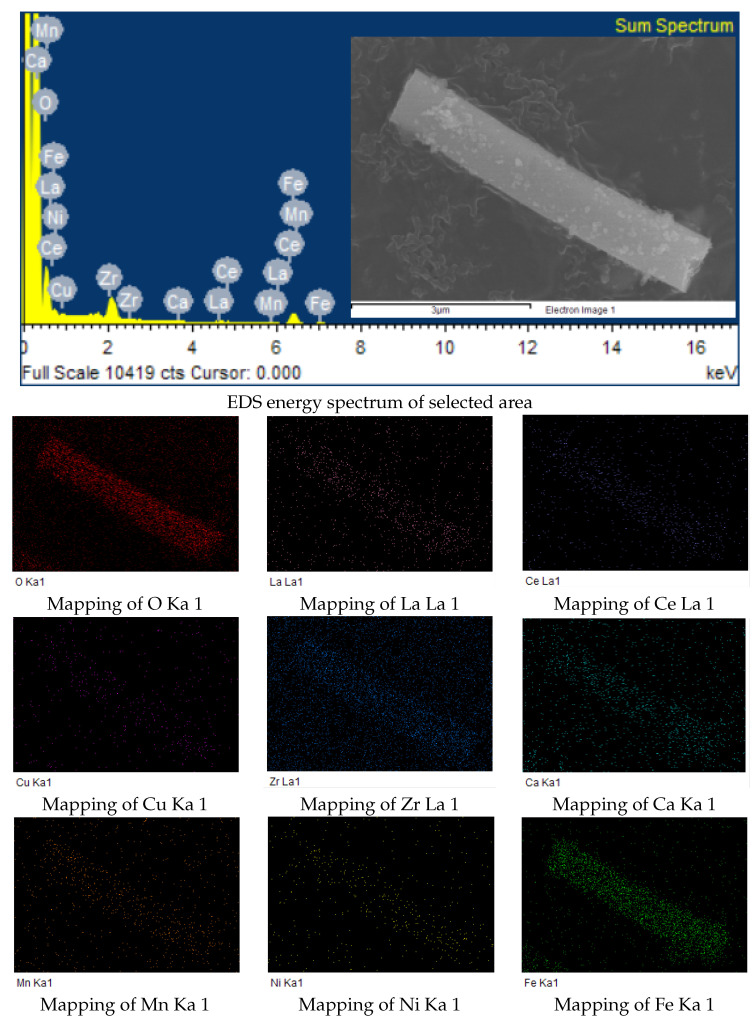
SEM and mapping of high entropy spinel ferrite nanofibers.

Figure 4 shows SEM images of high-entropy spinel ferrite nanofibers with a length of ∼5.78 μm and a diameter of ∼900 nm. EDS mapping (Figure 4) shows that the elements are uniformly distributed on the fiber surface.

The crystallographic surface index, half-height width, and grain size of the high-entropy spinel ferrite nanofibers were analyzed using Jade software. The main peak at 2θ was 35.6${}^{\circ }$, corresponding to the (311) plane, FWHM of 0.818${}^{\circ }$, and grain size of ∼10.3 nm. Additional characteristic peak parameters are listed in Table 1.

**Figure 5:**
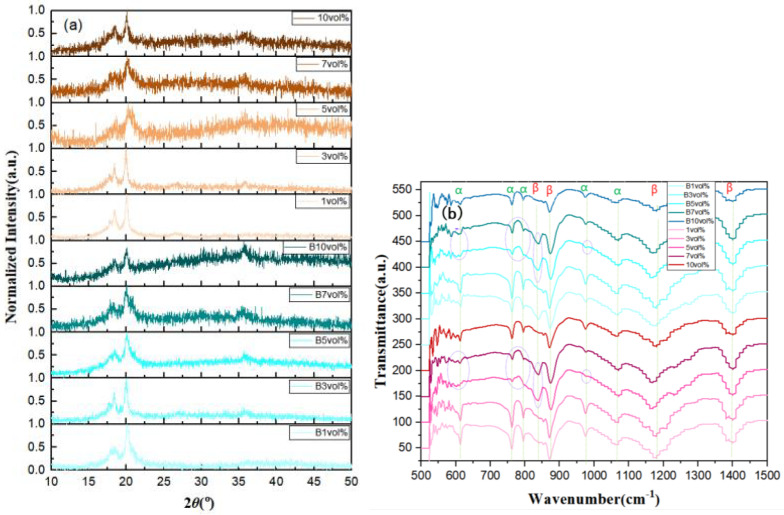
XRD (**a**) and FTIR (**b**) for 7FO/PVDF nanocomposite films with different doping ratios.

For characterization of the films, physical phase analysis of PVDF was conducted using XRD and FTIR, as shown in Figure 5a. The XRD patterns of 1 vol%, 3 vol%, 5 vol%, 7 vol%, and 10 vol% 7FO NF-doped PVDF films were treated by a magnetic field at 0.8 T for 3 min and without magnetic field (the films treated with the magnetic field are indicated as B in the picture). Different contents of high-entropy spinel ferrite and application of the magnetic field significantly affected the characteristic peaks of PVDF films with different phase contents. The characteristic peaks at 2θ were 17.72${}^{\circ }$, 18.39${}^{\circ }$, and 19.92${}^{\circ }$ for doping concentrations of 1 vol%, 3 vol%, and 10 vol%, respectively, for the films without magnetic field treatment, corresponding to the (100), (020), and (021) crystal planes of the α-phase. At 5 vol% and 7 vol% doping concentrations, the α-phase was suppressed and the 17.2${}^{\circ }$, 18.39${}^{\circ }$, and 19.92${}^{\circ }$ characteristic peaks gradually disappeared to 20.3${}^{\circ }$, corresponding to the (110) and (200) crystal planes of the β-phase. For the magnetic field-treated films, the characteristic peaks of the 1 vol% films contained 18.50${}^{\circ }$ and 20.03${}^{\circ }$. Three more characteristic peaks were restored with increasing doping concentration up to >7 vol%, when the 17.2${}^{\circ }$ and 18.39${}^{\circ }$ characteristic peaks were suppressed and the 20.03${}^{\circ }$ characteristic peak was dominant. The diffraction peak at 35.5${}^{\circ }$ corresponds to the main peak of high-entropy spinel ferrite oxide, which was more pronounced with increasing doping concentration and was enhanced by application of the magnetic field. There is no specific rule for the effect of the magnetic field on the characteristic peaks, and depending on the concentration of doping, there may be either suppression or promotion of the β-phase content.

Figure 5b displays the FTIR spectra of the α-phase of PVDF films. The α-phase peaks at 613, 764, 794, and 975 cm^−1^ gradually disappear and the β-phase at 840 cm^−1^ gradually increases when the doped high-entropy spinel ferrite content is 5 vol% or 7 vol%, regardless of whether or not the films were treated with a magnetic field. This can be attributed to the increase in dipole density increasing the β-phase content. According to the Beer–Lambert law, the relative fractions of β-phase in all crystalline phases can be determined from the absorption peaks of the α-phase and β-phase in the FTIR data results, for which the following equation can be used [39], as shown in Equation (6):

(6) $$F\left(\beta \right)=\frac{X_{\beta }}{X_{\alpha }+X_{\beta }}=\frac{A_{\beta }}{\left(K_{\beta }/K_{\alpha }\right)A_{\alpha }+A_{\beta }}=\frac{A_{\beta }}{1.26A_{\alpha }+A_{\beta }}$$

Here, $X_{\alpha }$ and $X_{\beta }$ in Equation (6) denote the mass fractions of the α and β phases, respectively, $A_{\alpha }$ and $A_{\beta }$ denote the absorbance of the materials at 764 and 840 cm^−1^, respectively, and $K_{\alpha }$ (6.1 × 10${}^{4}$ cm^2^/mol) and $K_{\beta }$ (7.7 × 10${}^{4}$ cm^2^/mol) represent the absorption coefficients of the α and β phases, respectively [40]. The calculated β-phase content of the ten samples of 1 vol%, 3 vol%, 5 vol%, 7 vol%, 10 vol%, B 1 vol%, B 3 vol%, B 5 vol%, B 7 vol%, and B 10 vol% were 47%, 50%, 31%, 33%, 47%, 37%, 51%, 33%, 36%, and 46%, respectively. Among these, 3 vol% 7FO/PVDF nanocomposites with 51% β-phase content was treated for 3 min under a 0.8 T uniform magnetic field.

**Figure 6:**
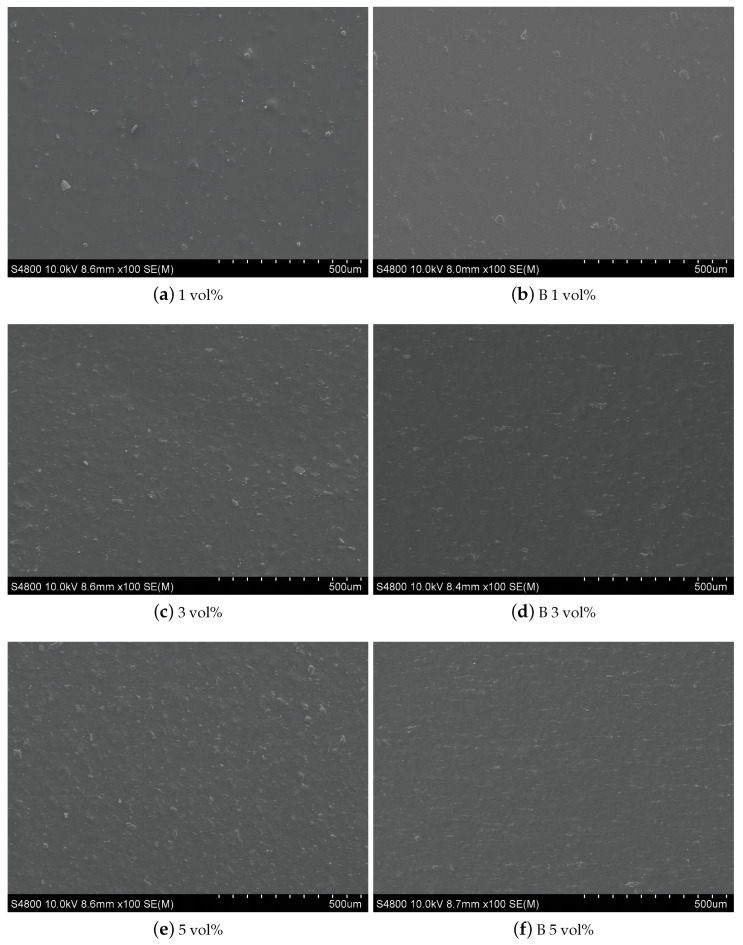

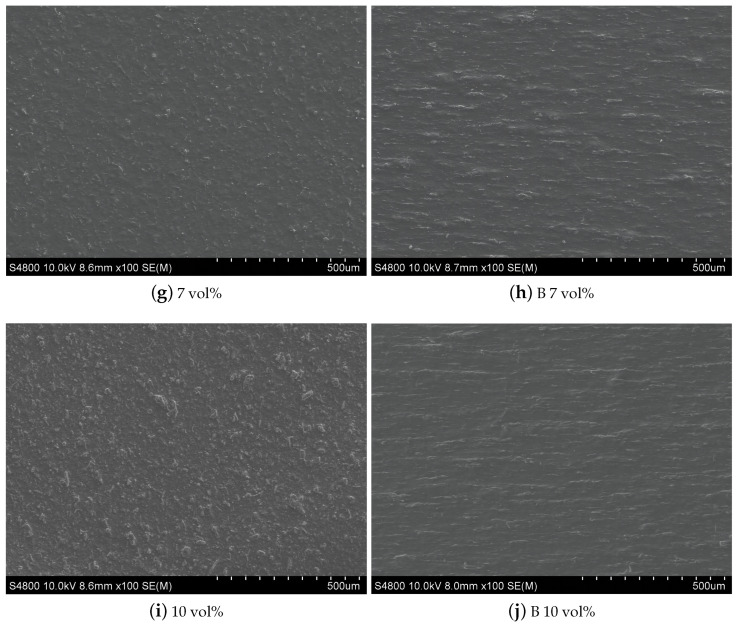
SEM images of 7FO/PVDF nanocomposite films with different doping ratios.

The microstructure of the composite film surface was observed by SEM (see Figure 6), and it can be seen that the fibers on the film surface become more dense with increasing doping concentrations of high-entropy spinel ferrite. portion of the ferrite behaved in a non-fibrousmanner; this may be related to conditional factors such as the humidity of the spinning conditions and long spinning time, resulting in the adhesion of the fibers into a block. However, this does not affect the material composition. The composite films were driven by the magnetic field. The originally uniformly distributed non-oriented nanofibers became oriented end to end in the magnetic field direction, forming parallel chains with each other, and the doping concentration increased this effect.

**Figure 7:**
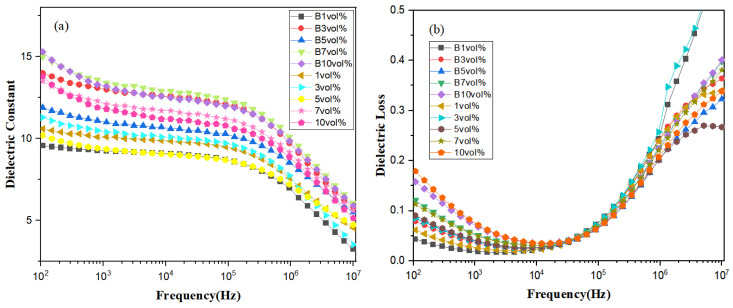
Dielectric constant (**a**) and dielectric loss (**b**) for 7FO/PVDF nanocomposite films with different doping ratios.

The effect of magnetic field induction on the dielectric properties of PVDF-based nanocomposites doped with different ratios of high-entropy spinel ferrite (La_0.14_Ce_0.14_Mn_0.14_Zr_0.14_Cu_0.14_Ca_0.14_Ni_0.14_)Fe_2_O_4_ NFs is an essential topic to explore. XRD and FTIR studies confirmed the evolution of the magnetic field versus the doping concentration on the β-phase in 7FO/PVDF nanocomposites. According to the dielectric frequency mapping in Figure 7a, the top three films with high dielectric constants were all PVDF-based composite films treated by the magnetic field. This indicates that the magnetic field promoted the interaction of high-entropy spinel ferrite with the PVDF matrix, which increases the dipole concentration and enhances the dielectric constant. In addition, we found an interesting variation in the permittivity rise-fall-rise-fall permittivity for the films treated with the magnetic field (or not). The final law, namely, the magnitude of the dielectric constant for both schemes, showed 7 vol% > 10 vol% > 3 vol% > other low contents. Although most of the literature on PVDF-based nanocomposites doped with high dielectric constant ceramics suggests that the doping concentration is positively correlated with a high dielectric constant, a few studies have reported [41,42,43] that it does not satisfy the positive correlation at certain frequencies.

The reasons for the high dielectric constants are as follows:

i. The 7FO nanofiber surfaces are uniformly charged in PVDF-based nanocomposite films. These charges may interact with opposite charges in the polymer chains and generate additional dipoles, contributing to the increase in the dielectric constant [44].

ii. The interaction force between the magnetic field and the dipole is keener, which may produce more dipoles, thereby contributing to the net dipole moment and increasing the dielectric constant.

Therefore, when the content is B 7 vol%, the film has the maximum dielectric constant of 13.45 at 1 kHz. However, a magnetic field of 0.8 T induced for 3 min had the greatest effect on the content of the 3 vol% composite film with the strongest enhancement of the dielectric constant.

Figure 7b shows the dielectric loss curves of the films. First, the loss of both schemes increased slowly with the increase in the doping concentration of high-entropy spinel ferrite. This is due to the formation of many interfaces in the nanocomposite film, in turn leading to additional dipole relaxation and increasing the tangential loss [45]. In addition, as the doping concentration rises, the high entropy spinel ferrite is more prone to agglomeration in PVDF substrates, which causes more loss. Second, at higher frequencies, the loss increases significantly due to the competing relaxation phenomenon of the crystalline phases of PVDF and 7FO nanofibers [46]. Third, the composite films with the same doping concentration slightly suppress the loss when using magnetic field treatment. In summary, the B 1 vol% magnetic field treatment had the least loss (0.0188).

Increasing breakdown strength is another a key method for boosting energy storage performance. The breakdown strength of composite films was analyzed based on a two-parameter Weibull statistical function, as shown in Equation (7):

(7) $$P\left(E\right)=1-exp{\left(\frac{E}{E_{b}}\right)}^{\beta }$$

where P(E) is the aggregate probability of electrical malfunctions, *E* is the experimental breakdown strength, $E_{b}$ refers to the breakdown strength with an aggregate failure probability of 63.2% (the Weibull breakdown strength), and β is the Weibull parameter associated with the reliability of the film under the electric field.

**Figure 8:**
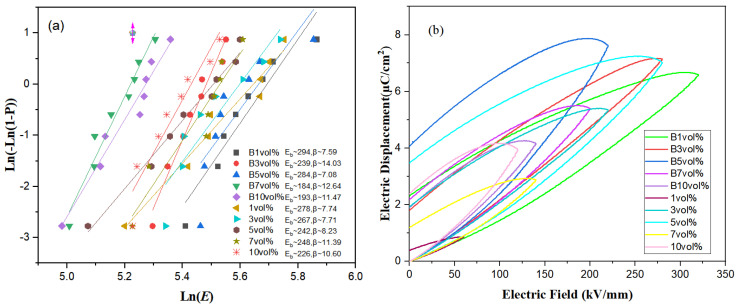
Breakdown strength (**a**) and D-E curves (**b**) for 7FO/PVDF nanocomposite films with different doping ratios.

Figure 8a shows the characteristic breakdown intensities of ten thin film samples and their β parameters. It can be seen that B 1 vol% 7FO/PVDF had a maximum breakdown strength of 294 kV/mm and B 3 vol% 7FO/PVDF had a maximum Weibull parameter of 14.03. The influences on breakdown strength include both intrinsic and extrinsic factors. The intrinsic factors are related to the band gap of the material, while the extrinsic factors are related to mechanical electrical breakdown due to over-limiting deformation caused by surface-bound charges and electrostriction effects, as well as to thermal breakdown due to heat and dielectric losses generated by leakage current exceeding its dissipation capacity [7,47].

Figure 8b shows the monopolar displacements with electric field loops of 7FO/PVDF nanocomposite films with different doping ratios under magnetic field-induced and non-magnetic field-induced conditions. The magnetic field-treated B 1 vol%, B 3 vol%, B 5 vol%, B 7 vol%, and B 10 vol% nanocomposite films had higher saturation polarization values (Ps) of ∼6.60 μC/cm^2^ (320 kV/mm), 7.05 μC/cm^2^ (280 kV/mm), 7.63 μC/cm^2^ (220 kV/mm), 5.40 μC/cm^2^ (200 kV/mm), and 4.17 μC/cm^2^ (140 kV/mm), respectively. These values were significantly higher than for the PVDF-based nanocomposite films without magnetic field treatment, which were ∼0.85 μC/cm^2^ (60 kV/mm), 5.35 μC/cm^2^ (220 kV/mm), 7.02 μC/cm^2^ (280 kV/mm), 2.88 μC/cm^2^ (140 kV/mm), and 3.99 μC/cm^2^ (119 kV/mm), respectively. The top four large saturation polarizations were B 1 vol%, B 3 vol%, B 5 vol%, and 5 vol%, with B 3 vol% having a slightly smaller residual polarization value of ∼1.8 μC/cm^2^. The magnetic field had a beneficial effect on the saturation polarization and a suppressive effect on the residual polarization. Thus, the high saturation polarization and low residual polarization observed in magnetic field-induced 3 vol% ((La_0.14_Ce_0.14_Mn_0.14_Zr_0.14_Cu_0.14_Ca_0.14_Ni_0.14_)Fe_2_O_4_ PVDF films has excellent energy density and higher efficiency. According to the above-mentioned law, B 7 vol% has the maximum dielectric constant of 13.45 at a frequency of 1 kHz.

**Figure 9:**
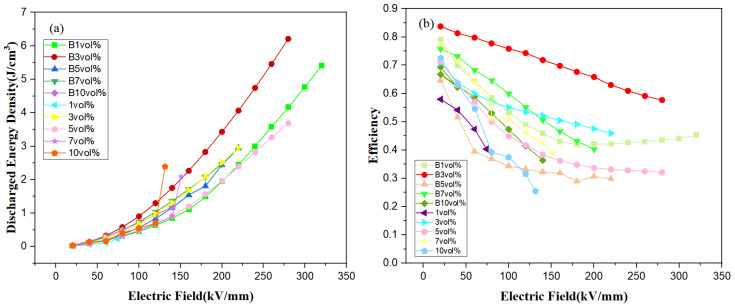
Discharge energy density (**a**) and efficiency (**b**) for 7FO/PVDF nanocomposite films with different doping ratios.

Figure 9a shows the discharge energy density of magnetic field-induced and non-induced 7FO/PVDF nanocomposite film materials, which can be calculated from the D-E hysteresis line according to Equation (1). The two samples with the largest discharge energy densities among the ten different films were B 1 vol% (5.41 J/cm^3^, 320 kV/mm) and B 3 vol% (6.23 J/cm^3^ 280 kV/mm), both treated with the magnetic field. The overall discharge energy density of the magnetic field-treated films was larger than those without magnetic field treatment for the same doping ratio, indicating that the magnetic field enhanced the breakdown voltage, which in turn significantly increased the discharge energy density.

Figure 9b shows the efficiency of each film; interestingly, the variation law is consistent with the second variation law of the dielectric constant. The discharge efficiency of the composite films with or without magnetic field treatment followed the same ascending-descending-ascending-descending variation law during the high-entropy spinel doping process, with concentrations from 1 vol% to 10 vol%. This indicates that the dielectric constant of the dielectric material has a certain connection with the discharge efficiency. The overall efficiency of the magnetic field-treated composite film was higher as well. The 3 vol% (La_0.14_Ce_0.14_Mn_0.14_Zr_0.14_Cu_0.14_Ca_0.14_Ni_0.14_)Fe_2_O_4_ PVDF film treated with a 0.8 T magnetic field for 3 min had the maximum discharge energy density, with 6.23 J/cm^3^ at 280 KV/mm voltage for 58% efficiency.

## 4. Conclusions

In this work, high-entropy spinel ferrite was used as an effective polymer nucleating agent to increase the β-phase of PVDF. We then applied a magnetic field to change the distribution morphology of the spinel ferrite filler in the PVDF. According to the SEM results, ferrite is initially distributed uniformly in the form of aggregation, then aggregated ferrite nanofibers are transformed into a connected chain by the magnetic field force under the application of the magnetic field. According to the XRD and FTIR results, a phase change occurs in the PVDF under doping and magnetic field treatment, with the maximum β-phase content being 51% due to the elevated dipole content. The dielectric and ferroelectric properties of the composites were investigated, and the results show that the magnetic field and (La_0.14_Ce_0.14_Mn_0.14_Zr_0.14_Cu_0.14_Ca_0.14_Ni_0.14_)Fe_2_O_4_ have a positive effect on the dielectric constant, loss, discharge energy density, and efficiency of the PVDF-based composite materials. The composite film with 3 vol% 7FO/PVDF and application of the magnetic field showed a significant enhancement in dielectric constant and a slight decrease in loss, an improvement in breakdown performance, an increase in dipole density, a larger potential shift polarization, and a smaller residual polarization, leading to its having a maximum discharge energy density of 6.23 J/cm^3^ at 275 kV/mm and an efficiency of 58%.

This work is of high research significance. First, high-entropy spinel ferrite can be utilized in catalysis, piezoelectricity, pyroelectricity, dielectric energy storage, lithium ion batteries, supercapacitors, and more. Second, the technical route of this experiment provides ideas for the path or treatment of the development of dielectric polymer materials, lithium battery electrodes, and other materials, which could optimize their related properties.

## Figures and Tables

**Table 1:** The crystal plane index, half-width, and grain size of the sample obtained with JADE software.

Number	hkl	FWHM	D/(Å = 0.1 nm)
1	220	0.629	132
2	311	0.818	103
3	400	0.651	133
4	511	0.583	158
5	440	0.810	116

## Data Availability

Data is contained within the article. The data presented in this study are available in this paper.
